# The Time-Course of Antioxidant Irisin Activity: Role of the Nrf2/HO-1/HMGB1 Axis

**DOI:** 10.3390/antiox10010088

**Published:** 2021-01-11

**Authors:** Agnieszka Irena Mazur-Bialy, Ewa Pocheć

**Affiliations:** 1 Department of Biomechanics and Kinesiology, Institute of Physiotherapy, Faculty of Health Science, Jagiellonian University Medical College, Grzegorzecka 20, 31-531 Krakow, Poland; 2 Department of Glycoconjugate Biochemistry, Institute of Zoology and Biomedical Research, Faculty of Biology, Jagiellonian University, Gronostajowa 9, 30-387 Krakow, Poland; ewa.pochec@uj.edu.pl

**Keywords:** irisin, inflammation, antioxidant, cytoprotection, macrophages

## Abstract

The production of free radicals is one of the basic mechanisms giving rise to the antimicrobial activity of macrophages; however, excessive accumulation of reactive oxygen species (ROS) can lead to cell damage, cell death, and release of the highly proinflammatory alarmin high-mobility group box 1 (HMGB1). This study aimed to evaluate the kinetics of antioxidant properties of the adipomyokine irisin administered shortly before or after macrophage activation to assess its effect on the nuclear factor erythroid 2-related factor 2 (Nrf2)/heme oxygenase-1 (HO-1)/HMGB1 pathway. The studies were performed on RAW 264.7 mouse macrophages treated with irisin (0, 25, and 50 nM) 2 h before or after lipopolysaccharide (LPS) stimulation. The effectiveness of respiratory burst and the expression of key factors of the antioxidant pathway, such as HO-1, Nrf2, superoxide dismutase 1 (SOD-1), SOD-2, glutathione peroxidase (GPx), catalase-9 (Cat-9), and HMGB1, were assessed. Irisin (50 nM) effectively reduced the free-radical production by macrophages. Furthermore, in both models, irisin altered the kinetics of expression of key factors of the downstream Nrf2/HO-1/HMGB1 pathway, leading to the increased production of Nrf2 and HO-1 and significantly reduced expression and release of HMGB1. In conclusion, irisin is a modulator of the Nrf2/HO-1/HMGB1 pathway and shows antioxidative and anti-inflammatory effects when administered both before and shortly after the activation of inflammatory mechanisms in mouse macrophages.

## 1. Introduction

Macrophages are the main cells of the innate immune system responsible for phagocytosis and the killing of microorganisms as a primary line of immune defense [1]. Generation of mitochondrial reactive oxygen species (ROS) by reduced nicotinamide adenine dinucleotide phosphate (NADPH) oxidase in activated macrophages during the process, named respiratory or oxygen burst, is a major defense mechanism against pathogens [2]. ROS are produced continuously in mitochondria during the respiratory electron transport chain in all aerobic cells [3,4]. Activation of macrophages by various signals, including pathogen-derived associated molecular patterns (PAMPs, e.g., bacterial lipopolysaccharide, LPS) accelerates mitochondrial oxygen consumption, as well as ROS generation and release [2,5,6]. ROS are crucial to macrophage effector functions and the elimination of pathogens [2]. However, the excessive accumulation of ROS can exert oxidative damage of the main cellular macromolecules (proteins, lipids, and DNA) and results in the necrosis of cells [7].

To control ROS accumulation and avoid cell destruction, an enzymatic antioxidant system is activated as a primary line of antioxidant defense. Antioxidant enzymes, such as superoxide dismutases (SODs), catalases (Cats), and glutathione peroxidases (GPxs), catalyze reactions aimed at the elimination of redundant ROS [8]. In addition to antioxidant enzymes, nonenzymatic endogenous and exogenous molecules with antioxidant activity support cellular defense [9]. The mechanisms of antioxidant activity include a reduction in ROS production, direct scavenging of ROS, and an impact of antioxidants on ROS-degrading pathways [6]. Antioxidant enzymes and other antioxidant compounds act synergistically and interdependently to protect cells from the damage caused by ROS [10].

Our previous studies demonstrated that irisin (IR), a myokine discovered in 2012, shows antioxidant activity against oxidative burst generated by macrophages in response to lipopolysaccharide (LPS) stimulation when used 24 h earlier [11,12]. The results of multidirectional studies have demonstrated that irisin, a cytokine secreted by skeletal muscle cells during physical activity, not only regulates browning of white adipose tissue, as described primarily, but has a much wider spectrum of activity both in physiological (neurogenesis, bone physiology) and in pathological (inflammation, obesity, carcinogenesis) states [13]. Our research on the role of irisin in innate immune response showed its anti-inflammatory activity on nonstimulated and LPS-stimulated murine RAW 264.7 macrophages when applied 24 h before stimulation [11,14], as well as on adipocytes, the main nonimmunocompetent cells in adipose tissue, pre-incubated with IR before LPS stimulation [15,16].

Our present research is in line with the search for therapeutic antioxidants among molecules naturally produced by mammalian cells to control respiratory burst in macrophage inflammation. Monitoring of oxidative stress generated by macrophages, characteristic for the pathogenesis of numerous disorders, including common bacterial infections and more serious cardiovascular and neurological diseases, by natural antioxidants is an important goal of antioxidant therapies [17]. The aim of the present study was to assess the antioxidant potential of irisin on the inflammatory activation of macrophages when administered 2 h before induction of macrophage activation by LPS (pre-incubation) and 2 h after LPS-stimulation (post-incubation). To achieve this goal, we analyzed the time-course gene and protein expression of nuclear factor erythroid 2-related factor 2 (Nrf2) and heme oxygenase-1 (HO-1) cytoprotective antioxidant proteins, the messenger RNA (mRNA) expression of SOD-1, SOD-2, Cat-9, and GPx antioxidant enzymes, and the gene expression and release of the high-mobility group box 1 (HMGB1) late mediator of inflammation, for detailed analysis of irisin’s effects on the downstream Nrf2/HO-1/HMGB1 pathway, all in the presence of two irisin concentrations (25 and 50 nM) applied in the pre- and post-incubation schemes.

## 2. Materials and Methods

### 2.1. Cell Culture and Experimental Design

**Figure 1:**
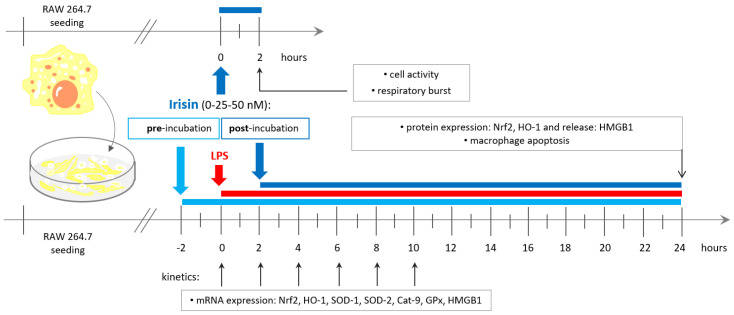
Schematic diagram representing the timelines for irisin administration to RAW 264.7 macrophages. The cells were stimulated with lipopolysaccharide (LPS; 100 ng/mL), treated with irisin (0, 25, or 50 nM) 2 h before (pre-incubation) and 2 h after (post-incubation) LPS activation, and harvested at six time points (0, 2, 4, 6, 8, or 10 h) for the gene expression studies or 24 h for the analysis of protein expression and release, as well as the determination of macrophage apoptosis. Overall activity and respiratory burst were also assessed after 2 h incubation of irisin with RAW 264.7 cells. Nrf2: nuclear factor erythroid 2-related factor 2; HO-1: heme oxygenase-1; SOD-1: superoxide dismutase 1; SOD-2: superoxide dismutase 2; Cat-9: catalase 9; GPx: glutathione peroxidases; HMGB1: high-mobility group box 1.

The study was conducted on a RAW 264.7 macrophage cell line derived from male Balb/c mice. The cell line was purchased from the European Type Culture Collection (ETCC, Sigma-Aldrich, St. Louis, MO, USA) and was *Mycoplasma*-tested. For the experiment, cells were maintained under standard conditions (37 °C, 5% CO_2_) in Dulbecco’s modified Eagle medium (DMEM) supplemented with 10% fetal bovine serum (FBS), and 1% antibiotics in the presence of nonglycosylated, recombinant irisin (IR; 0, 25, and 50 nM; Cayman Chemicals, Ann Arbor, MI, USA); some of the cells were stimulated with LPS (100 ng/mL; *Escherichia coli*, serotype 0111: B4, Sigma-Aldrich, St. Louis, MO, USA) for the next 2, 4, 6, 8, or 10 h (gene expression studies) or 24 h (protein expression and release studies). We used the nonglycosylated type of irisin in accordance with our previous study where we showed that nonglycosylated irisin exerts a more prominent effect than its glycosylated counterpart [16]. Moreover, the intention of our research was to determine the effect of pharmacological IR concentrations and to maintain continuity with our previous studies in which we used 0, 25, and 50 nM irisin, as well as a higher and effective concentration of 100 nM [11,14]. After incubation, supernatants and cell pellets were frozen (−60 °C) for future quantification of protein and mRNA levels. The timeline scheme of irisin administration is presented in Figure 1.

### 2.2. Cell Activity and Respiratory Burst Generation

Overall cell viability and activity were determined after 2 h of incubation with various irisin concentrations (0, 25, or 50 nM) using a commercial CellTiter kit (Promega, Madison, WI, USA) according to the manufacturer’s instructions. Respiratory burst generation (production of intracellular free radicals) was measured using a nitroblue tetrazolium salt (NBT; Sigma-Aldrich, St. Louis, MO, USA) with PMA (phorbol 12-myristate 13-acetate, 1 μg/mL; Sigma-Aldrich, St. Louis, MO, USA) as an activator after 45 min of stimulation. For both tests, absorbance was measured using the spectrophotometer Expert Plus (ASYS/Hitech, Eugendorf, Austria).

### 2.3. Apoptosis Assay

To detect the percentage of apoptotic macrophages after 24 h of LPS stimulation and irisin treatment at two concentrations (25 or 50 nM) 2 h before LPS stimulation (pre-incubation) and 2 h after LPS stimulation (post-incubation), cells were stained with the Annexin V-PE Apoptosis Detection Kit I (BD Pharmingen, San Diego, CA, USA) according to the manufacturer’s protocol. The percentage of apoptotic cells was quantified by flow cytometry (FACSCaliburTM; BD Biosciences, San Diego, CA, USA).

### 2.4. Quantitative Real-Time PCR (qPCR)

Total RNA was extracted using the RNeasy Plus Mini Kit (Qiagen, Hilden, Germany) following the manufacturer’s instructions. RNA concentration was measured using NanoDrop 2000 (Thermo Scientific, Rockford, IL, USA), and mRNA (1 μg) was reverse-transcribed into complementary DNA (cDNA) using the High-Capacity cDNA Reverse Transcription Kit (Thermostat plus, Eppendorf, Hamburg, Germany) according to the manufacturer’s protocol. Gene expression was quantified by qPCR using SybrGreen Gene Expression Master Mix (Applied Biosystems, Austin, TX, USA) and primers for SOD-1, SOD-2, GPx, Cat-9, Nrf2, HO-1, and HMGB1 in the StepOnePlus Real-Time PCR System (Applied Biosystems, Foster City, CA, USA). Glyceraldehyde phosphate dehydrogenase (GAPDH) served as the endogenous control. The sequences of the primers are available in our previous paper [12]. The expression of analyzed genes was normalized to GAPDH expression (RQ) using the 2^−ΔΔCt^ method.

### 2.5. Western Blot Analysis of Nrf2 and HO-1 Protein Expression

Nfr2 and HO-1 cytoplasmatic protein content in the RAW 264.7 cell line cultured with different IR concentrations was analyzed by Western blotting. Moreover, A549 cells overexpressing Nrf2 were used as a positive control. Human endothelium (hE) with Nrf2 gene silencing or mouse induced pluripotent stem cells (miPSC) with HO-1 gene knockout were used as negative controls. The cells were lysed by sonication in radioimmunoprecipitation assay (RIPA) buffer (Thermo Scientific, 89900, Rockford, IL, USA) with a protease inhibitor cocktail (Sigma-Aldrich, P2714, St. Louis, MO, USA) and centrifuged (18,000 rpm) for 20 min at 4 °C. The protein concentration was measured using the Total Protein Kit, Micro Lowry, Peterson’s Modification (Sigma-Aldrich, TP0300, St. Louis, MO, USA). Equal amounts of proteins (30 μg) in Laemmli Sample Buffer (Bio-Rad, Hercules, CA, USA) with β-mercaptoethanol were boiled for 5 min and separated on 4–20% Tris–glycine SDS-PAGE gels (Bio-Rad, 456-1094, CA, USA), before being transferred to a polyvinylidene fluoride (PVDF) membrane (Thermo Scientific, Rockford, IL, USA). After blocking in 5% skim milk in Tris-buffered saline/Tween-20 (TBST) (Nrf2, HO-1; 2 h) or 1% bovine serum albumin (BSA) in TBST (Actb, overnight), the PVDF membranes were immunodetected using the appropriate primary antibodies, rabbit polyclonal anti-Nrf2 (Proteintech, 16396-1-AP, 1:2000, Chicago, IL, USA) and rabbit polyclonal anti-HO-1 (Enzo Life Sciences, ADI-SPA-894, 1:500, Farmingdale, NY, USA), both diluted in 5% skim milk in TBST (overnight), as well as mouse monoclonal anti-actin β (Actb, Thermo Fisher Scientific, MA5-15739, Rockford, IL, USA) diluted 1:4000 in 1% BSA in TBST (2 h). Then, the blots were washed three times with TBST, and the secondary sheep anti-rabbit IgG (AP322A) and goat anti-mouse (Sigma-Aldrich, A1682, St. Louis, MO, USA) antibodies conjugated with alkaline phosphatase (AP) were applied in a 1:4000 dilution for 1 h at room temperature (RT). The proteins were visualized by a colorimetric reaction using 5-bromo-4-chloro-3-indolyl phosphate (BCIP) and NBT substrates (Roche Molecular Biochemical, Mannheim, Germany) for AP. Molecular weights of Nrf2 and HO-1 were verified using a Page Ruler Prestained Protein Ladder (Thermo Scientific, 26616, Rockford, IL, USA). The intensity of Nrf2 and HO-1 bands was measured densitometrically (UVI Map, UVItec, Cambridge, UK) and normalized to the intensity of Actb used as a loading control.

### 2.6. Examination of HMGB1 Release

After 24 h of incubation, the supernatants were collected, and the level of HMGB1 was quantified using a commercial ELISA Kit according to the manufacturer’s instructions, measured using an Expert Plus spectrophotometer (ASYS/Hitech, Eugendorf, Austria).

### 2.7. Statistical Analysis

Data were tested for normality of distribution, and differences among groups were determined using ANOVA with Tukey post hoc analysis. All data were expressed as means ± standard deviation (X ± SD) of four or five independent experiments with the level of statistical significance (*p*) set at 0.05.

## 3. Results

### 3.1. Irisin Attenuates Respiratory Burst Generated by Macrophages and Their Apoptosis

**Figure 2:**
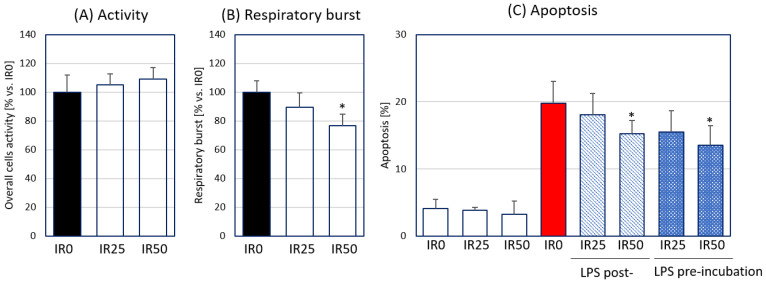
The effect of irisin (IR; 0, 25, or 50 nM) on RAW 264.7 macrophages in terms of overall activity and viability: (**A**) cell activity measured using the CellTiter test; (**B**) respiratory burst generation by macrophages stimulated with phorbol 12-myristate 13-acetate (PMA; free-radical production) measured 45 min after PMA stimulation; (**C**) apoptosis intensity measured with the Annexin V kit 24 h after lipopolysaccharide (LPS; 100 ng/mL) stimulation in the presence of IR administered 2 h before and 2 h after LPS treatment. Results are presented as the percentage vs. the IR0 + LPS group with standard deviation; *n* = 4–5. Statistically significant differences were calculated in relation to the IR0 + LPS group, used as a control group: * *p* < 0.05.

Firstly, we determined the impact of IR used at two concentrations (25 or 50 nM) on the overall activity of RAW 264.7 macrophages via an evaluation of the cell activity, production of reactive oxygen species (ROS) by PMA-activated macrophages, and apoptosis of RAW 264.7 cells. The activity of macrophages determined using the CellTiter test was not changed in the presence of either IR concentration (Figure 2A). The assessment of oxidative burst by IR-treated macrophages in response to PMA showed a significant decrease in intracellular ROS generation in the presence of the highest IR concentration (50 nM) (Figure 2B). Irisin at this concentration also reduced apoptosis of the activated RAW 264.7 cells when applied 2 h before and 2 h after LPS stimulation (Figure 2C). In sum, IR at a concentration of 50 nM was able to suppress the ROS generation by activated macrophages and the apoptosis of these phagocytes stimulated by bacterial LPS.

### 3.2. Irisin Effects on Nrf2 and HO-1 Gene and Protein Expression

**Figure 3:**
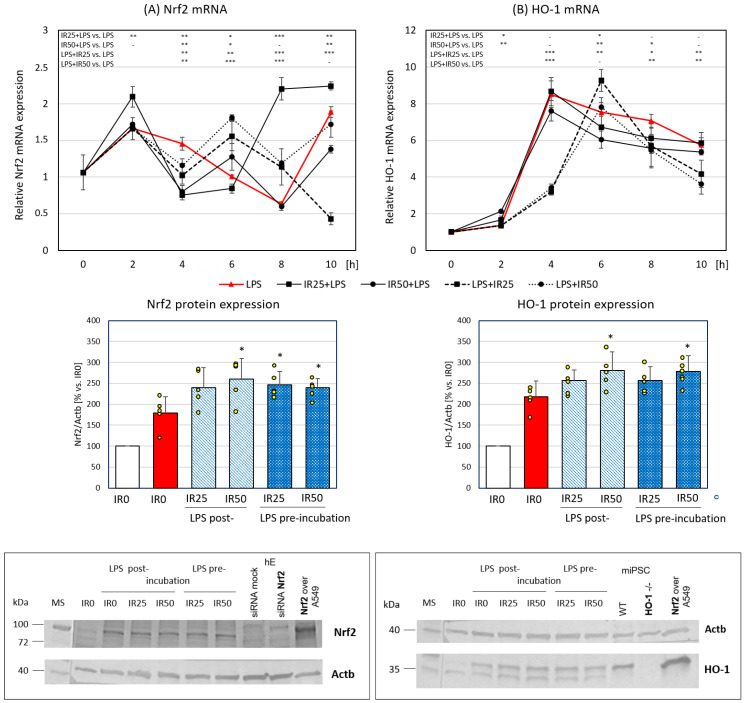
The time-course of nuclear factor erythroid 2-related factor 2 (Nrf2) and heme oxygenase-1 (HO-1) expression in RAW 264.7 macrophages after irisin (IR; 0, 25, or 50 nM) administered before or after LPS treatment: (**A**) kinetics of Nrf2 messenger RNA (mRNA) expression (upper panel) and Nrf2 protein expression 24 h after lipopolysaccharide (LPS; 100 ng/mL) stimulation (lower panel); (**B**) kinetics of HO-1 mRNA expression (upper panel) and HO-1 protein expression 24 h after LPS stimulation (lower panel); irisin was administered 2 h before (IR + LPS) or after LPS treatment (LPS + IR). Negative controls: siRNA mock and siRNA Nrf2 (hE: human endothelium; for Nrf2); HO-1 -/-: HO-1 gene knockout (miPSC: mouse induced pluripotent stem cells). Positive control: Nrf2 overexpression in A549 cells (Nrf2 over). Results are presented as the mean with standard deviation; *n* = 5. Statistically significant differences were calculated in relation to the IR0 + LPS group, used as a control group: * *p* < 0.05; ** *p* < 0.01; *** *p* < 0.001; “-“ not statistically significant. Actb: actin β; MS: mass standard; WT: wild type.

In the next step of our study, we focused on the Nrf2 transcription factor and HO-1, both known as antioxidative and anti-inflammatory factors. The activity of Nrf2 is crucial for protection against oxidative stress, as well as the attenuation of inflammation generated by macrophages [18]. To determine the suppression of inflammation by IR, we analyzed Nrf2 expression at six time points after macrophage stimulation by LPS (0, 2, 4, 6, 8, and 10 h). LPS, a glycolipid abundantly present in the outer membrane of Gram-negative bacteria, is widely used to trigger innate immunity via the activation of professional phagocytes, including macrophages [4,19]. The time-course of *Nrf2* expression in the activated RAW 264.7 macrophages in the presence of IR was a dynamic process dependent on the IR concentration and the application scheme. The highest enhancement of *Nrf2* gene expression was observed in RAW 264.7 cells pretreated with IR at the concentration of 25 nM. In this variant, *Nrf2* gene expression was significantly increased shortly (2 h) after macrophage activation and at the latest time points (8 and 10 h) in comparison to nontreated cells. The remaining IR variants (50 nM in the pre-incubation scheme and both IR concentrations in the post-incubation scheme) effectively raised *Nrf2* mRNA expression 6 h after LPS stimulation. The analysis of protein level showed a statistically significant increase in Nrf2 content in RAW 264.7 macrophages pre-incubated with IR50 and post-incubated with both IR doses (25 and 50 nM). These results corresponding to the increase in *Nrf2* gene expression observed 6 h after LPS stimulation (Figure 3A). 

HO-1, in addition to its primary function in heme homeostasis, was shown to be important for antioxidant protection and the anti-inflammatory response in macrophages [17]. HO-1 gene expression grew significantly in RAW 264.7 macrophages 4 h after LPS stimulation (control group). Pre-incubation with IR increased *HO-1* mRNA levels as early as 2 h after macrophage activation in an IR dose-dependent manner up to double the control level. In the case of post-incubation, the effect of enhanced *HO-1* expression was shifted to 6 h after LPS stimulation and was induced only by the lower IR concentration. Similar to *Nrf2* expression, the *HO-1* mRNA level was reduced in IR-treated macrophages 4 h after LPS stimulation. At the later points of the time-course analysis, both concentrations of IR post-incubated with RAW 264.7 cells attenuated *HO-1* gene expression. The analysis of protein content showed a statistically significant enhancement of HO-1 expression in the presence of both IR50 administered 2 h before and IR50 administered 2 h after LPS stimulation (Figure 3B). The increase in HO-1 level by 50 nM IR was deemed dependent of Nrf2 early regulation because the expression of Nrf2 was affected by this IR dose (Figure 3A).

In sum, IR had an enhancing effect on the antioxidant defense Nrf2/HO-1 axis in macrophages in a dose- and time-dependent manner.

### 3.3. Irisin Effects on the Expression of Crucial Antioxidant Enzymes SOD-1, SOD-2, GPx, and Cat-9

ROS produced during an innate immune response to LPS are important in pathogen defense, but their excess can be harmful for macrophages [6]. Oxidative burst is controlled in activated macrophages by antioxidant enzymes such as superoxide dismutases (SODs), catalase 9 (Cat-9), and glutathione peroxidases (GPxs) which act sequentially to eliminate redundant ROS [8]. SODs catalyze the dismutation of superoxide (O_2_^•^^−^) into oxygen (O_2_) and hydrogen peroxide (H_2_O_2_), which is subsequently degraded by Cat-9 or GPx to H_2_O [20]. The mRNA expression of these antioxidant enzymes was analyzed in our study at six time points after LPS activation of RAW 264.7 macrophages (as for Nrf2 and HO-1 gene expression described in Section 3.2) to demonstrate the antioxidant activity of irisin toward the inflammation generated by macrophages.

**Figure 4:**
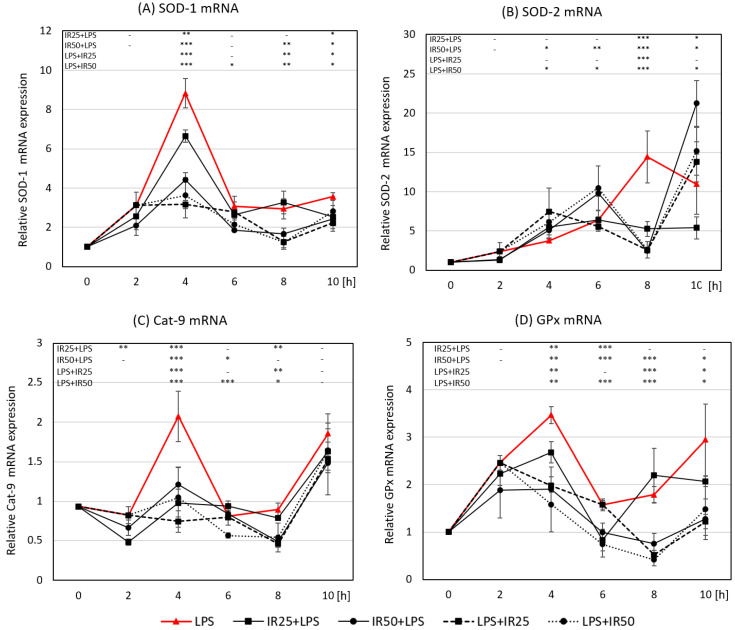
The time-course of the expression of crucial antioxidant enzymes in RAW 264.7 macrophages after irisin (IR; 0, 25, or 50 nM) before or after lipopolysaccharide (LPS; 100 ng/mL) treatment: (**A**) kinetics of superoxide dismutase 1 (*SOD-1*) mRNA expression; (**B**) kinetics of *SOD-2* mRNA expression; (**C**) kinetics of catalase-9 (*Cat-9*) mRNA expression; (**D**) kinetics of glutathione peroxidase (*GPx*) mRNA expression. Irisin was administered 2 h before (IR + LPS) or after LPS stimulation (LPS + IR). Results are presented as the mean with standard deviation; *n* = 4–5. Statistically significant differences were calculated in relation to the IR0 + LPS group, used as a control group: * *p* < 0.05; ** *p* < 0.01; *** *p* < 0.001; “-“ not statistically significant.

We assessed the gene expression of two SOD metalloproteinases important in cellular oxygen metabolism: cytoplasmic Cu/Zn SOD-1 and mitochondrial Mn SOD-2. *SOD-1* gene expression was markedly increased in RAW 264.7 macrophages 4 h after LPS stimulation; then, between 6 and 10 h after activation, SOD-1 expression normalized to a level comparable to that seen in the first 2 h of the time-course study. At the peak of *SOD-1* gene transcription, IR added at both concentrations 2 h before phagocyte activation was much more effective in the reduction of *SOD-1* mRNA expression than IR administered 2 h after stimulation. The effect of *SOD-1* mRNA reduction exerted by post-incubated IR increased at 8 h of activation in contrast to the lower concentration of pre-incubated irisin (Figure 4A). At this point, it should be recalled that the intensity of oxygen burst measured with the NBT test 45 min after its administration showed a significantly lower intensity of respiratory burst in macrophages pre-incubated with IR (50 nM). The lower production of ROS in the IR50 group (Figure 2B) displayed, at the same time, a weaker signal for the activation of enzymes responsible for their elimination.

The time-course analysis of *SOD-2* showed a peak in gene expression later than that seen for the SOD-1 enzyme (at 8 h after LPS activation), while the effect of IR on this enzyme’s expression was also different than that seen for the *SOD-1* mRNA level. IR at a concentration of 50 nM used in both pre- and post-incubation increased *SOD-2* expression at 4, 6, and 10 h after macrophage stimulation in contrast to the lower IR dose, which was ineffective at these time points. It is worth noting that IR accelerated the initial peak of this gene activation, followed by a marked drop in expression at 8 h (Figure 4B).

The profile of *Cat-9* gene expression in the activated RAW 264.7 macrophages (control group) was similar to that seen for *SOD-1* up to 8 h after LPS stimulation, whereas, at the latest time point, *Cat-9* increased significantly, nearly to the level reached 6 h after activation. Regardless of the time of IR treatment and its concentration, the highest *Cat-9* gene expression reached 4 h after macrophage stimulation was significantly attenuated at the latest time points. A reduction in *Cat-9* expression was also observed 2 h after activation in macrophages pre-incubated with the lowest IR concentration (25 nM) and at the 6 h time point in both variants with the higher IR concentration (50 nM), as well as at the 8 h time point in all variants except for pre-incubation with the higher IR concentration (50 nM). The high *Cat-9* expression at the latest time point was not affected by IR (Figure 4C).

The glutathione peroxidase family contains seven members (GPx1–7) in mammalian cells, representing the most abundant oxidoreductases, i.e., the enzymes responsible for the oxidization of glutathione to glutathione disulfide and the reduction of H_2_O_2_ to H_2_O [3,21]. We observed the highest expression of GPx in RAW 264.7 macrophages 4 h after LPS stimulation. IR used in both pre-incubation and post-incubation schemes lowered the *GPx* mRNA level in a dose-dependent manner. A higher efficiency of GPx reduction was observed at the 4 h time point as a result of IR post-incubation (Figure 4D).

In sum, the expression of antioxidant enzymes increased as the result of macrophage activation. The time-course quantitative analysis of gene expression showed the highest level of mRNA for *SOD-1*, *Cat-9*, and *GPx* antioxidant enzymes 4 h after LPS stimulation of macrophages, while the mRNA level of *SOD-2* was most abundant 8 h after activation. Considering the lower production of ROS by macrophages treated with the higher IR concentration (50 nM), pre-incubation of macrophages with IR provoked significantly lower *SOD-1*, *Cat-9*, and *GPx* expression even 2 h after LPS stimulation, which was more prominent in the IR post-incubated model. In turn, *SOD-2* expression was enhanced 4 and 6 h after activation by 50 nM irisin administered both before and after LPS stimulation, and then significantly reduced at the 8 h time point when the level of *SOD-2* was highest in control cells.

### 3.4. Irisin Effects on HMGB1 Expression and Release

**Figure 5:**
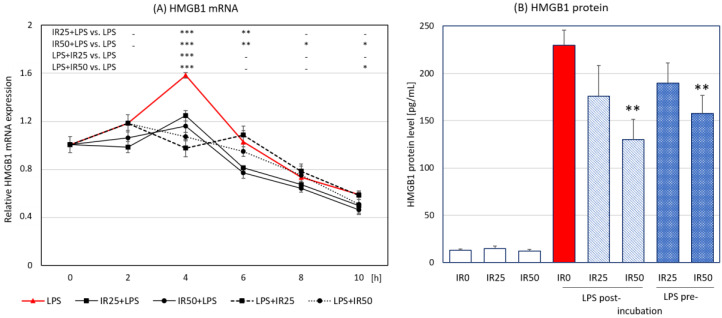
The time-course of high-mobility group box 1 (HMGB1) expression and release in LPS-stimulated RAW 264.7 macrophages after irisin (IR; 0, 25, or 50 nM) before or after lipopolysaccharide (LPS; 100 ng/mL) treatment: (**A**) kinetics of *HMGB1* mRNA expression and (**B**) HMGB1 protein expression released 24 h after LPS stimulation. Irisin was administered 2 h before (IR + LPS) or after LPS treatment (LPS + IR). Results are presented as the mean with standard deviation; *n* = 4–5. Statistically significant differences were calculated in relation to the IR0 + LPS group, used as a control group: * *p* < 0.05; ** *p* < 0.01; *** *p* < 0.001; “-“ not statistically significant.

High-mobility group box 1 (HMGB1) is a nuclear DNA-binding protein responsible for the regulation of gene transcription, and it was also shown to be secreted by activated macrophages into the extracellular matrix. HMGB1 is a late mediator of inflammation [22], which contributes to macrophage reprogramming toward the proinflammatory M1-like phenotype [23], and its expression is closely connected to and modulated by the Nrf2/HO-1 pathway [24]. The *HMGB1* gene expression analyzed in our study reached a peak 4 h after LPS stimulation of macrophages. The highest level of the *HMGB1* transcript was significantly diminished by IR applied both before and after LPS stimulation, while IR post-incubation exerted the more profound effect on *HMGB1* gene expression. The suppressing effect was extended to 6 h after LPS stimulation in the case of 25 nM IR and to 10 h after LPS stimulation when the higher concentration was applied (Figure 5A). After the initial decrease in *HMGB1* expression following the administration of IR, a slight increase could be observed, albeit not reaching the level observed in other groups. The ELISA assay showed the significant inhibitory impact of IR used at the higher concentration in both application schemes on HMGB1 secretion (Figure 5B). We demonstrate that the macrophage-derived HMGB1 was downregulated by IR administered both shortly before and after the activation of these cells.

## 4. Discussion

Oxidative stress is a state of imbalance between the production and neutralization of free radicals, resulting from the failure of antioxidant systems [25]. The key regulator of the response to oxidative stress is the Nrf2 transcription factor, which triggers a cascade of numerous antioxidant factors, including HO-1 [26]. Given that the induction of HO-1 by Nrf2 requires the activation of p38 mitogen-activated protein kinases, there is a strong association between inflammation and the activation of antioxidant mechanisms [27]. Moreover, oxidative stress itself can lead to the activation of inflammatory mechanisms, including the release of the highly proinflammatory alarmin HMGB1 [28]. Therefore, the role of the Nrf2/HO-1/HMGB1 pathway has received considerable attention in studies of the mechanisms of many diseases and pathologic states, especially in hypoxia-induced injury in the heart, intestine, or brain [29,30,31,32,33,34].

In the present study, we assessed the effect of short (2 h) pre- or post-incubation with IR on the activation of the Nrf2/HO-1/HMGB1 downstream pathway in LPS-stimulated macrophages. We aimed to observe how IR modulates the time-course of the expression of key antioxidant factors associated with them HMGB1. In our study, Nrf2 was significantly elevated at the protein level when cells were treated with both IR doses 2h after, and IR50 2 h before LPS administration. At the same time, a significantly weaker respiratory burst and ROS production was observed when cells were treated with IR50. At the same time, we observed a significant increase in HO-1 protein expression in IR50-treated groups which correspond with Nrf2 up-regulation. It should be noted that HO-1 expression is regulated by a number of factors. One of them is the release of nitric oxide related to the activity of iNOS (inducible nitric oxide synthase). Research by Alcaraz et al. showed that irisin modulates the NO release by macrophages, leading to a significant increase in concentration when administered after the inflammatory factor [35]. In our unpublished studies, we saw that IR administration 2 h after LPS induced a marked increase in iNOS expression between 4 and 6 h, lasting until 8 h before decreasing. This increase in iNOS expression, as well as the initiation of NO production, may be another factor activating the expression of HO-1 at later time points. The current research is in line with our earlier observations, where we showed that IR effectively inhibits the production of free radicals, leading to the overexpression of factors involved in the Nrf2/HO-1 pathway [12]. We also demonstrated IR’s anti-inflammatory activity which was associated with the activation of the Toll-like receptor 4 (TLR4)/myeloid differentiation primary response 88 (MyD88) pathway, leading to a reduction in the release of key inflammatory factors [11,14]. Both these observations were, however, obtained in a model where IR was administered 24 h before macrophage stimulation. Considering the short duration of IR activity, with a half-life of less than 1 h [36], the mechanisms observed in both models (administration of irisin 24 h before vs. 2 h before or after LPS stimulation) reflect the various physiological situations. Moreover, we have not yet assessed whether IR exerts antioxidative and anti-inflammatory effects when administered upon activation of the macrophage response to the pathogen or PAMPs (assessed after LPS stimulation in this study). The results of the present study showed that the antioxidative effect of IR is related to the activation of the Nrf2/HO-1 pathway, regardless of the time of its administration. However, an effective reduction in the release of ROS was observed only at the highest IR concentration (50 nM). Considering the significant effect of IR on reducing the respiratory burst and the subsequent release of oxygen radicals by macrophages, there was no intensification of effector mechanisms in the form of expression of key enzymes of the antioxidant pathway. Nevertheless, in both models, we observed earlier activation of *SOD-2* expression, with a decreased expression of *SOD-1*, *GPx*, and *Cat-9*; this finding may indicate a special role of SOD-2 in the regulation of antioxidant mechanisms by IR. In addition to our research on macrophages, the modulating effect of IR on antioxidant mechanisms was also demonstrated in a liver ischemia/reperfusion injury model [37], in a murine model of chronic pancreatitis [38], and in the protection against endothelial injury in arteriosclerosis [39]. Our research indicates that the mechanism of protective effects of IR may be at least partly related to the antioxidant mechanisms triggered in immunocompetent cells, and it can be assumed that IR’s antioxidant potential may be used in pharmacotherapy in the future.

Excessive production of ROS leads to cell damage through the oxidation of macromolecules and the activation of strong proinflammatory factors such as the alarmin HMGB1 [40]. HMGB1 is a molecule with pleiotropic action, and it is involved in both physiological and pathological processes. Its primary function is to stabilize the DNA structure; however, in a state of stress or inflammation, it can be secreted via an active or passive mechanism, acting extracellularly as a strong proinflammatory cytokine, thereby promoting an increase in late mortality due to sepsis or endotoxemia [40,41,42]. Previous research confirmed that the release of HMGB1 can be stimulated by excessive accumulation of ROS [28] and that the Nrf2/HO-1 pathway plays an important role in the regulation of HMGB1 release [24]. Upregulation of HO-1 inhibits the translocation of HMGB1 to the cytosol, thus reducing its active secretion [24,29]. On the other hand, studies on pulmonary fibrosis showed that downregulation of HMGB1 is necessary for effective antioxidant activity of Nrf2, which plays a pivotal role in HO-1 production [43]. In the present study, the level of HMGB1 expression and release was significantly downregulated by IR (50 nM) when administered 2 h before and after LPS stimulation. This is in line with the finding of our previous study wherein HMGB1 release was significantly reduced when IR was administered 24 h before macrophage activation, but a higher concentration (100 nM) was required to achieve the inhibitory effect [14]. Considering the abovementioned relationship between HMGB1 release and antioxidant status, the reduction in HMGB1 expression and release was influenced by both the reduced production of ROS by IR (50 nM)-treated macrophages and the increase in the expression of HO-1 proteins. However, in both cases, in terms of the antioxidant and anti-inflammatory effects of irisin, only the higher irisin concentration showed efficacy. These results suggest that IR may be an effective factor in combat against HMGB1-related pathologic conditions such as ischemia/reperfusion injury, arthritis, sepsis, or endotoxemia via regulation of the Nrf2/HO-1/HMGB1 pathway, thus leading to the activation of antioxidant mechanisms while inhibiting the release of proinflammatory HMGB1.

## 5. Conclusions

In conclusion, our research is the first study to show IR’s influence on the time-course of expression of key components involved in the Nrf2/HO-1/HMGB1 downstream pathway in LPS-activated RAW 264.7 macrophages. As demonstrated in our study, IR administered 2 h before or after stimulation enhanced the antioxidant mechanisms in activated macrophages. This enhancement was associated with the increased expression of Nrf2 and HO-1 and the significant reduction in effectiveness of the oxidative respiratory burst. As a consequence of reduced exposure to free radicals, the expression of key enzymes in the antioxidant pathway was not further enhanced. Moreover, IR reduced the overexpression of the alarmin HMGB1, dependent on the Nrf2/HO-1 pathway, thus exerting both antioxidative and anti-inflammatory effects.
